# Aortic Valve Replacement for Moderate Aortic Stenosis with Severe Calcification and Left Ventricualr Dysfunction—A Case Report and Review of the Literature

**DOI:** 10.3389/fcvm.2017.00014

**Published:** 2017-03-27

**Authors:** Nikhil Narang, Roberto M. Lang, Vladimir M. Liarski, Valluvan Jeevanandam, Marion A. Hofmann Bowman

**Affiliations:** ^1^Department of Medicine, Section of Cardiology, University of Chicago Medical Center, Chicago, IL, USA; ^2^Department of Medicine, Section of Rheumatology, University of Chicago Medical Center, Chicago, IL, USA; ^3^Department of Surgery, University of Chicago Medical Center, Chicago, IL, USA

**Keywords:** calcific aortic valve disease, aortic stenosis, aortic valve replacement, rheumatoid arthritis, heart failure

## Abstract

A 55-year-old man with a history of erosive, seropositive rheumatoid arthritis (RA), and interstitial lung disease presented with shortness of breath. Echocardiography showed new-onset severe left ventricular (LV) dysfunction with an ejection fraction (EF) of 15% and moderately increased mean aortic valve gradient of 20 mmHg in a trileaflet aortic valve with severe sclero-calcific degeneration. Coronary angiography revealed no significant obstructive coronary disease. Invasive hemodynamic studies and dobutamine stress echocardiography were consistent with moderate aortic stenosis. Guideline directed medical therapy for heart failure with reduced EF was initiated; however, diuretics and neurohormonal blockade (beta-blocker and angiotensin receptor blocker) provided minimal improvement, and the patient remained functionally limited. Of interest, echocardiography performed 1 year prior to his presentation showed normal LV EF and mild aortic leaflet calcification with moderate stenosis, suggesting a rapid progressing of calcific aortic valve disease. Subsequently, the patient underwent surgical aortic valve replacement and demonstrated excellent postsurgical recovery of LV EF (55%). Calcific aortic valve disease is commonly associated with aging, bicuspid aortic valve, and chronic kidney disease. Pathophysiological mechanism for valvular calcification is incompletely understood but include osteogenic transformation of valvular interstitial cells mediated by local and systemic inflammatory processes. Several rheumatologic diseases including RA are associated with premature atherosclerosis and arterial calcification, and we speculated a similar role of RA accelerating calcific aortic valve disease. We present a case of accelerated aortic valve calcification with (only) moderate stenosis, complicated by a rapid decline in LV systolic performance. Guidelines for AVR in moderate stenosis without concomitant cardiac surgery are not well established, although it should be considered in selected patients.

## Introduction

A 55-year-old man with a medical history significant for erosive, seropositive rheumatoid arthritis (RA), and interstitial lung disease (ILD) was hospitalized for shortness of breath and bilateral foot pain over the past 3 weeks. He described worsening pain in the bilateral medial malleoli and both great toes similar to pain he had in the past related to his diagnosis of RA. He described becoming more dyspneic on exertion than his baseline, for which he required supplemental oxygen. Initial vital signs on presentation were as followed: blood pressure of 146/106 mmHg, heart rate 124 beats per minute and regular, respiratory rate of 28 breaths per minute, body mass index 34.5 kg/m^2^, and pulse oximetry was 92% on 3 L supplemental oxygen by nasal cannula. The patient was alert and had mild respiratory distress on presentation. Cardiac examination was notable for a soft systolic murmur on the left sternal border, jugular venous distension to 15 mmHg above the clavicle, and fine inspiratory crackles at the bases of both lungs. Skin was warm to touch with 2+ lower extremity edema bilaterally from the mid-shin downward. Initial laboratory values were notable for sodium of 133 mmol/L, potassium 4 mmol/L, creatinine 1.3 mg/dL, and an elevated N-terminal pro b-type natriuretic peptide of 1,829 pg/mL (Table 1). LDL was 119 mg/dL, HDL 56 mg/dL, trigylcerides 166 mg/dL, glucose 91 mg/dL, and HbA1c 5.5%. White blood cell count and hemoglobin were within normal limits at 6.3 × 10^3^/μL and 15.7 g/dL, respectively. The electrocardiogram showed sinus tachycardia, and no ST and T wave changes, concerning for acute ischemia. Prior to admission, medications included albuterol-ipratroprium 18–103 μg 2 puffs four times per day, carvedilol 6.25 mg twice a day, famotidine 20 mg twice a day, furosemide 20 mg once daily, certolizumab injection once per month, and hydroxychloroquine 200 mg twice a day.

**Table 1:** **Serial measurements of pro-BNP**.

Date	Pro-BNP (reference range <125 pg/mL)
03/2014	63
12/2014	234
03/2015	548
04/2015	852
11/2015	1,520
11/2015	1,829
12/2015	1,027
03/2016	2,035
→7/2016 surgical aortic valve replacement	
11/2016	239

Chest X-ray revealed moderate cardiomegaly with bibasilar consolidations and moderate pulmonary edema with small pleural effusions bilaterally. Bilateral foot X-rays revealed chronic erosions at the head of the left great toe and mild soft tissue swelling and a chronic appearing erosion of the proximal phalanx of the right great toe, both unchanged from prior study. His foot pain responded to analgesics.

The clinical presentation was suggestive of acute decompensated heart failure. This was confirmed with the transthoracic echocardiogram that demonstrated severely decreased left ventricular (LV) function with an ejection fraction (EF) of 10–15%, moderate mitral regurgitation, severe right ventricular dysfunction, and severe tricuspid regurgitation. The severity of AS could not be assessed due to image acquisition quality. Of note, an echocardiogram performed 11 months earlier had a normal EF of 55%, mild LV hypertrophy, normal right ventricular performance and size, moderate aortic valve sclero-calcific changes with a mean systolic trans-aortic gradient of 20 mmHg, and a calculated aortic valve area by continuity equation of 1.2 cm^2^, consistent with moderate AS. No other valvular lesions were present at that time.

For further evaluation of possible underlying causes for the new onset of biventricular dysfunction, a cardiac magnetic resonance imaging with vasodilator stress and gadolinium contrast was performed. Late gadolinium enhancement was present in the basal and mid-ventricular septum in a pattern consistent with a dilated cardiomyopathy and not a myocardial infarction. No perfusion defects were noticed. Further workup revealed distal segmental right lower lobe pulmonary embolus and he was appropriately started on rivaroxaban for pulmonary embolus. Other medical therapy for decompensated heart failure included diuresis with intravenous furosemide, followed with transition to discharge medication including lisinopril 20 mg daily, spironolactone 25 mg daily, carvedilol XL 25 mg daily, and furosemide 40 mg daily. A 4-day course of prednisone 10 mg was added in addition to home hydroxylchlorquine 200 mg twice daily for his history of RA. Furthermore, certolizumab doses were held due to development of heart failure.

The patient’s heart failure was medically optimized in follow-up consultations in our outpatient cardiology clinic, though he continued to demonstrate New York Heart Association Class III symptoms despite up-titration of goal-directed therapies of beta-blockers, angiotensin-converting enzyme inhibitors, aldosterone antagonists, and diuretics. It was postulated that his RA-related ILD, arterial hypertension, as well as suboptimal compliance with medications and diet were contributing to his ongoing dyspnea with minimal exertion.

Four months following his initial hospitalization and diagnosis of biventricular dysfunction and pulmonary embolism, the patient was re-hospitalized and admitted to the cardiac intensive care unit with respiratory distress requiring non-invasive positive pressure ventilation. Respiratory panel testing during workup revealed positive testing for *Influenza A* virus. He was treated with oseltamivir and briefly required inotropic therapy in addition to continuous furosemide infusion. Repeat echocardiogram showed again severely reduced EF (<20%) now with qualitatively severe calcific changes of the aortic valve with a mean gradient of 17 mmHg and an aortic valve area of 0.83 cm^2^. EKG showed sinus tachycardia at 123 bpm but was otherwise unremarkable. Right and left heart catheterization was performed for further evaluation of cardiogenic shock. Coronary angiogram revealed no significant obstructive coronary artery disease. Invasive hemodynamic measurements during right and left heart catheterization demonstrated increased biventricular filling pressures (RA 12 mmHg, RV 52/12 mmHg, PA 50/30 with mean of 23 mmHg, and LVEDP 30 mmHg), mildly reduced cardiac output (PA saturation 73%, cardiac index by Fick 2.9 L/min, and cardiac index by thermodilution 2.6 L/min), and moderate aortic stenosis with peak-to-peak pressure gradient of 20 mmHg between left ventricle and ascending aorta, with a calculated aortic valve area of 1.5 cm^2^ by the Hakki equation (1). The patient subsequently recovered from cardiogenic shock exacerbated by influenza A virus and was discharged from the hospital on medical therapy with low doses of metoprolol, lisinopril, and furosemide. In addition, he was continued on anticoagulation for his previously diagnosed subsegmental pulmonary embolus and hydroxychloroquine for RA.

The next encounter was 3 weeks later in the outpatient cardiology clinic when the patient presented in compensated and stable chronic heart failure NYHA class III. He was in no distress, normotensive with some findings consistent with heart failure including elevated jugular vein distension 10 cm above the clavicle, fine inspiratory crackles bilaterally, and 1+ edema bilateral above the ankle. A dobutamine stress echocardiogram was performed to further characterize the severity of calcific aortic valve disease. With dobutamine (20 μg/kg/min), the visual estimated EF increased from 25 to 43%, and this was associated with a change of mean baseline aortic gradient of 12–19 mmHg, with a calculated aortic valve area by the continuity equation of 1.37 cm^2^. These findings are consistent with moderately severe aortic stenosis and moderate LV dysfunction with good contractile reserve. Given the patient’s ongoing dyspnea and functional limitations, which had progressed over a year, along with new LV dysfunction without alternate cause, referral for aortic valve replacement was made. His case was discussed in an interdisciplinary colloquium including general cardiology, rheumatology, pulmonology, interventional cardiology, and cardiothoracic surgery. CT-chest and pulmonary function testing were obtained for preoperative risk stratification. The results of the pulmonary function testing were as follows: TLC 3.81 L (=57% of predicted), FVC 1.88 L (=42% of predicted), FEV1 1.55 L (=48% of predicted), FEV1/FVC 82% (=114% of predicted), and DLCO 8.78 (=32% of predicted) and were interpreted as moderately severe restrictive lung disease with a disproportionate reduction in diffusing capacity. The patient subsequently underwent surgical AVR with a 27-mm bioprosthetic aortic valve and tricuspid valve annuloplasty repair, and the initial post-op recovery was excellent. Patient’s echocardiogram post aortic valve replacement revealed recovery of EF to 55% (Table 2). However, on postoperative day 7, he developed signs of decreased end-organ perfusion and was found to have pericardial tamponade for which he underwent a pericardial window procedure, which drained 360 mL blood from the pericardium. There were no other complications, and patient was discharged to subacute rehabilitation facility 12 days after cardiac surgery. He was seen 6 weeks later in the cardiology clinic and was found to be in atrial flutter with 2:1 conduction and a heart rate of 126 bpm. This was associated with only minimal symptoms of slightly increased dyspnea on excertion. He underwent DC cardioversion and has been doing very well since then with documented sinus rhythm 2 and 3 months later.

**Table 2:** **Diagnostic testing in evaluation of aortic stenosis**.

Test	Interpretation of the study	Mean transaortic gradient (mmHg) and aortic valve area (cm^2^)	Ejection fraction (%)
06/2013	Moderate sclerocalcific changes No AS, no AI		49
Echocardiography			
03/2014	Moderate sclerocalcific changes	11 mmHg 1.6 cm^2^	53
Echocardiography			
12/2014	Moderate sclerocalcific changes Mild-moderate AS	19.5 mmHg 1.2 cm^2^	55
Echocardiography			
11/2015	Aortic stenosis, the severity of which cannot be accurately evaluated in the presence of such profound left ventricular (LV) systolic dysfunction		10–15
Echocardiography			
First presentation for new-onset heart failure			
03/2016	Technically difficult study LV moderately severe reduced Severe sclerocalcific changes	16.6 mmHg 0.83 cm^2^	
Echocardiography			
Presentation in cardiogenic shock, influenza A virus			
03/2016	Moderate AS Biventricular elevated filling pressures No obstructive CAD	LV/Aorta peak-to-peak gradient: 20 mmHg 1.5 cm^2^	
LHC and RHC			
Presentation in cardiogenic shock, influenza A virus			
05/2016 Dobutamine stress ECHO	Good contractile reserve Fixed moderate AS Baseline global hypokinesis with augmentation of all segments with stress	Gradient: 12 mmHg and increase to 19 mmHg	25 and increase to 43
		Area: 1.37/1.36 cm^2^	
7/2016	Severe aortic sclerosis with preserved leaflet opening. There is moderate aortic stenosis. There is trace aortic regurgitation.		
TEE-intraoperative during AVR			
7/2016	Bioprosthetic AVR Normal LV function		50–55
ECHO, POD 4			

*Patient had several echocardiograms performed between 2010 and 2016 due to his interstitial lung disease and chronic shortness of breath*.

## Discussion

Aortic valve disorders are the most common valvular lesions requiring surgical intervention. Aortic valve replacement is commonly utilized for symptomatic patients with severe aortic stenosis. In the setting of aortic stenosis, maintenance of LV systolic function is achieved by a compensatory increase in LV wall thickness to balance the pressure overload from the obstructive valvular lesion. As the degree of valvular obstruction progresses, the compensatory mechanism of LV hypertrophy is overwhelmed by the underlying wall stress, and LV dilation and a fall in EF, along with the development of symptoms, occur (2). We speculate that in patients with underlying subclinical cardiomyopathies, compensatory mechanisms will be overwhelmed at even less than severe AS. However, the role of AVR in symptomatic patients with moderate AS is controversial, and selection of the best approach remains clinically challenging usually due to the presence of multiple confounding comorbidities (3). The role for AVR in documented low-flow, low-gradient aortic stenosis, classified as moderate in severity is not well defined (4). The current case is an example of aortic stenosis consistently characterized as “moderate AS” by echocardiography and invasive hemodynamics, but also with accelerated calcification of the aortic valve leaflets and rapid decline of LV function. We have stepwise documentation of rapidly declining LV systolic function from a normal EF to one of 15% within 1 year. The underlying mechanisms leading to the encountered severe LV dysfunction are likely multifactorial and possibly include increased afterload from moderate aortic stenosis, along with potential some cardiotoxic side effects of conventional and biologic disease modifying antirheumatic medications, although this is controversial (5, 6). Importantly, the profound LV dysfunction was completely reversed within days of valve replacement emphasizing the significant role of afterload reduction for the treatment of a failing left ventricle, which is commonly seen in patients with severe AS and LV dysfunction (7, 8).

Among patients with a failing left ventricle, relief of a fixed afterload reduction in the form of significant aortic stenosis may result in the greatest long-term survival benefit. The current American Heart Association valvular heart disease guidelines give a Grade IIb recommendation stating that it is reasonable to perform aortic valve replacement for moderate AS in patients who are undergoing other cardiac surgeries. There are no guideline recommendations to guide treatment of symptomatic patients with reduced LV systolic function and moderate AS. However, a contemporary study by Samad and colleagues sought to assess the effect of AVR with and without coronary artery bypass surgery and survival in patients with moderate and severe AS. Of the 1,634 patients included in this retrospective single-center analysis, 1,090 of those were classified as moderate by valve area of >1 cm^2^ and mean gradient range of 25–39 mmHg. In a subgroup of those who underwent surgical AVR with a diagnosis of moderate AS, 135 underwent isolated AVR, while 152 had AVR plus coronary artery bypass surgery. In 135 patients who only had AVR, valve replacement within 90 days of index echocardiogram in the setting of moderate aortic stenosis and LV dysfunction (defined as <50%, 108 patients with EF<35%) conferred a significant long-term mortality benefit of 42% compared to those who did not undergo surgery (9). Although this was not a randomized clinical trial, the data suggest that AVR for moderate AS with LV dysfunction resulted in superior outcome over medical therapy in *selected* patients. Prior detriments to referring patients with symptomatic AS for valve replacement may include advanced age with short life expectancy, LV dysfunction, lung disease, and renal disease (10, 11). These data suggest that in the setting of LV dysfunction with moderate AS, as a contributor or bystander condition, relief of the hemodynamic burden of additional afterload likely contributes a significant survival benefit in selected patients. In the last decade, transcatheter aortic valve replacement (TAVR) has become the preferred treatment option for patients with severe AS and high operative risk (12). Recent trial data have even suggested that TAVR is non-inferior to surgical AVR in patients in intermediate-risk severe AS (13). Coexisting moderate AS and LV systolic dysfunction is not uncommon, and now with the advent of TAVR, upcoming clinical trials will seek to address whether a less invasive approach such as TAVR may be a viable strategy to reduce afterload in patient’s with LV systolic dysfunction and concomitant moderate aortic stenosis, who are of high surgical risk (14).

Our patient did not have baseline chronic kidney disease, diabetes, abnormalities in calcium and phosphorus serum concentrations, or advanced age, which are all common risk factors associated with calcific aortic valve disease. The underlying pathophysiology of accelerated calcification in patients with RA is poorly understood with a paucity of literature or guidelines to guide management. Although data on valvular disease are scarce, there is published literature on increased rates of coronary artery calcification in this population, which may represent a shared etiology (15–18). Whether this is related to autoimmune disease severity over time or is increased in all patients with RA is a matter of continuing investigation. However, it is plausible that vascular inflammation may contribute to both valvular and coronary artery changes by the same common mechanisms. Research now implicates osteogenic processes as key mechanism in calcific aortic valve disease and thereby providing two general directions for development of new therapies (1): inhibition of procalcific regulators and (2) induction of proresorptive regulators in aortic valve tissue (19, 20). However, tissue targeting may be essential to avoid damage to skeletal bone for example.

Calcific aortic valve disease commonly occurs in patients with bicuspid aortic valves (BAV). These patients present for AVR at a younger age and often have no concommittant obstructive coronary artery disease. It is estimated that up to 50% of patients requiring surgical AVR for severe aortic stenosis have BAV. Hemodynamic and developmental abnormalities of the congenital bicuspid aortic valve may predispose to premature calcification and valve failure, although no specific mechanisms have been reported. In contrast, patients with trileaflet aortic valve with severe calcification and stenosis present later in life for AVR (one decade later compared to BAV) and commonly share atherosclerotic risk factors, such as diabetes, chronic kidney disease, and increased levels of oxidized LDL. Up to 60% of patients with trileaflet AV and severe stenosis require coronary artery bypass grafting for obstructive coronary artery disease, which is only required in up to 10% in patients with BAV and severe stenosis (21). However, cellular mechanisms leading to calcific aortic valve disease remain incompletely understood, and as demonstrated in this case, our patient has a trileaflet aortic valve, no coronary artery disease, and no other atherosclerotic risk factors beyond RA.

## Concluding Remarks

Taken together, the current guidelines do not support routine AVR in moderate AS not undergoing concomitant cardiac surgery. Severe calcification alone in mild and moderate aortic stenosis independently predicts the rate of disease progression and survival in these populations (20). Careful evaluation for other causes of LV dysfunction and symptoms should be undertaken in patients who do not meet standard criteria for AVR. In these patients with progressive symptoms and no other clear explanation, the correction of a hemodynamic burden such as a calcified valve, even if only moderately severe when measured by mean aortic valve gradient, should be considered.

## Ethics Statement

This research was approved by the University of Chicago Institutional Review Board. Further written consent for publication of this case report was obtained from the patient.

## Author Contributions

All authors contributed to the analysis and interpretation of data, wrote the manuscript, approved the final version of the manuscript, and agreed to be accountable for all aspects of the work.

## Conflict of Interest Statement

The authors declare that the research was conducted in the absence of any commercial or financial relationships that could be construed as a potential conflict of interest.
